# Supplementary material for: The role of CXCR3/LRP1 cross-talk in the invasion of primary brain tumors
Source: Nat Commun. 2017 Nov 17;8:1571. doi: 10.1038/s41467-017-01686-y (PMC5691136; doi:10.1038/s41467-017-01686-y)
Supplement: Supplementary file 2 — Supplementary information [file 41467_2017_1686_MOESM2_ESM.pdf]

SUPPLEMENTARY FIGURES

A

Cell line	Grade	Histology	Reference
1321N1	II/III	Astrocytoma	Ponten J. et al (2)
NHATS	II/III	IHA	Sasai K. et al. (3)
NHATSR	II/III	IHA	Sasai K. et al. (3)
U87	IV	Glioblastoma	Ponten J. et al.; Allen M. et al. (1,2)
U118	IV	Glioblastoma	Ponten J. et al. (2)
T98G	IV	Gliobastoma	Stein GH. (4)

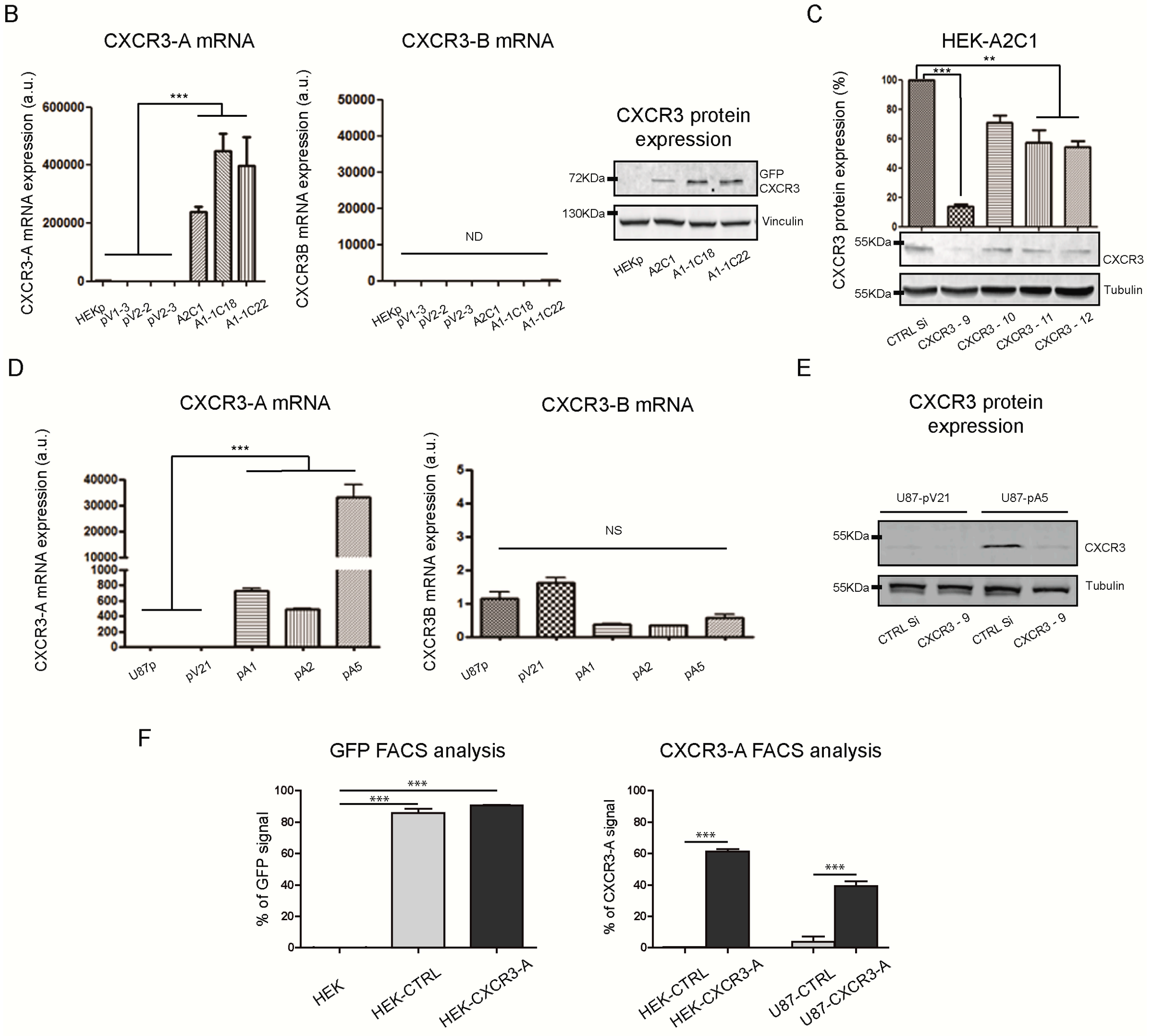

Supplementary Fig.1: CXCR3-A expression in stably transfected cells

(A) Glioma cell lines description and references[1-4]. (B) CXCR3-A (left panel) and CXCR3-B (middle panel) mRNA expression, performed by real time PCR in parental HEK-293 cells (HEKp), in HEK-CTRL (clones pV1-3, pV2-2 and pV2-3) and in HEK-CXCR3-A (clones A2C1, A1-1C18 and A1-1C22). Values were normalized to reference gene. CXCR3-A protein expression (right panel) were performed in parental HEK-293 and HEK-CXCR3-A clones by western blot using GFP antibodies. Vinculin was used as a loading control. (C) Silencing of CXCR3 in HEK-CXCR3-A cells using 4 different siRNA against

human CXCR3 (siCXCR3-9, -10, -11 and -12). Tubulin was used as a loading control. (D) Analyses of relative mRNA expression of CXCR3-A (left panel) and of CXCR3-B (right panel) were assessed by real time PCR in parental U87 cells, U87-CTRL cells (U87 pV21) and U87-CXCR3-A (U87 pA1, pA2 and pA5). Values were normalized to reference gene. (E) CXCR3 protein expression was performed by Western blot for CXCR3 in cell extracts from U87 pV21 and U87 pA5. Silencing of CXCR3 expression was performed using the most efficient siRNA for human CXCR3 (siCXCR3-9). Tubulin was used as a loading control. (F) FACS analysis was performed to measure GFP intensity (left panel) or CXCR3 intensity at the cell membrane (right panel) in HEK-CTRL and U87-CTRL or in HEK-CXCR3-A and U87-CXCR3-A. All the results from three independent experiments were combined to calculate mean and SEM, and values were normalized to those obtained for the control, *** P<0.001, ** P<0.01, a.u. for arbitrary unit. Multiple comparisons were performed with one-way analysis of variance, followed by Bonferroni post hoc tests.

A

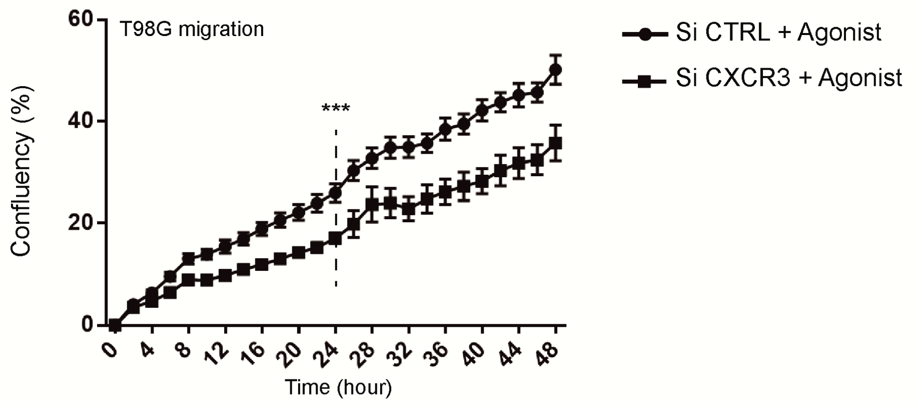

B

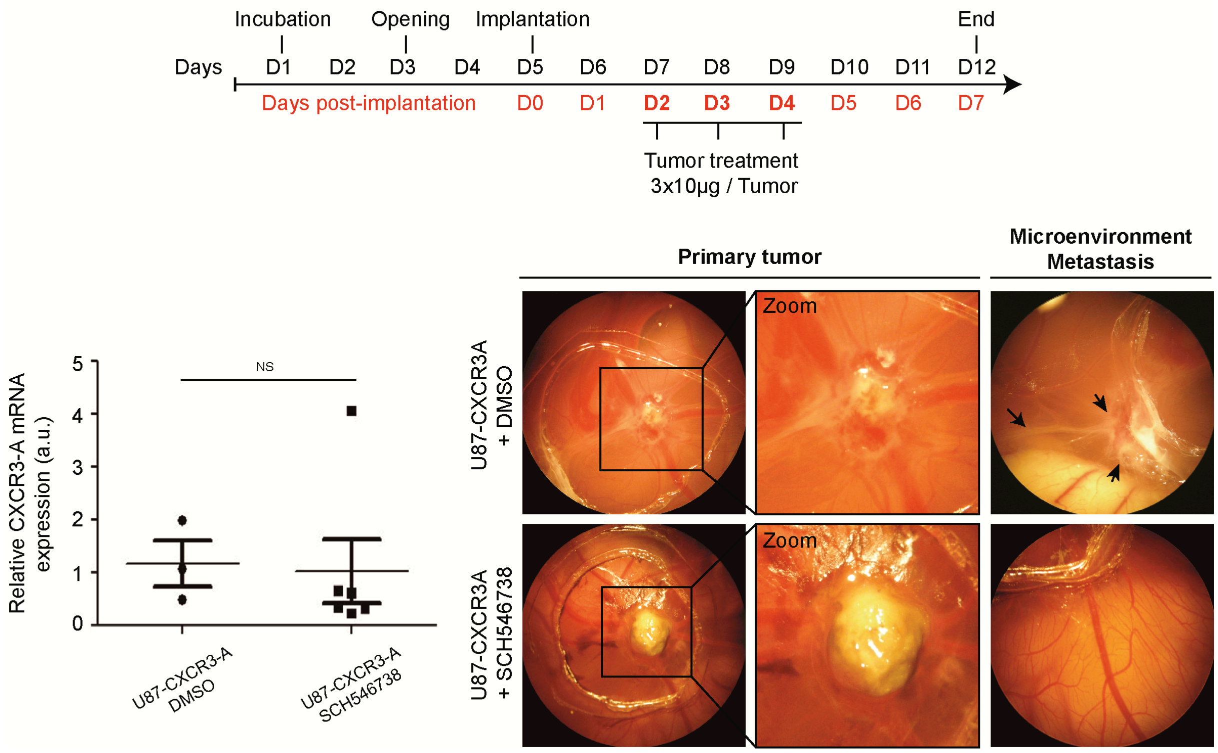

Supplementary Fig.2: Impact of CXCR3 silencing and inhibition in tumor cell migration and invasion
(A) *In vitro* effect of CXCR3 silencing. Cell migration assay (n=3) of T98G cells silenced for CXCR3 (Si CXCR3) versus control Si RNA (Si CTRL) stimulated with the agonist of CXCR3 (PS372424). *** P<0.001
(B) *In vivo* effect of CXCR3 inhibition. CAM assays were performed using U87-CXCR3-A cells treated with DMSO (negative control) or CXCR3 antagonist SCH546738 (n= 50 eggs for each condition). Seven days after implantation, relative CXCR3-A mRNA expression in U87-CXCR3-A tumors treated with DMSO or SCH546738 and images of CAM tumors were performed. Values were normalized to reference gene and to the DMSO control treatment. a.u.: arbitrary unit. NS: No significant difference. Statistical comparison between two groups was performed using the Mann-Withney test.

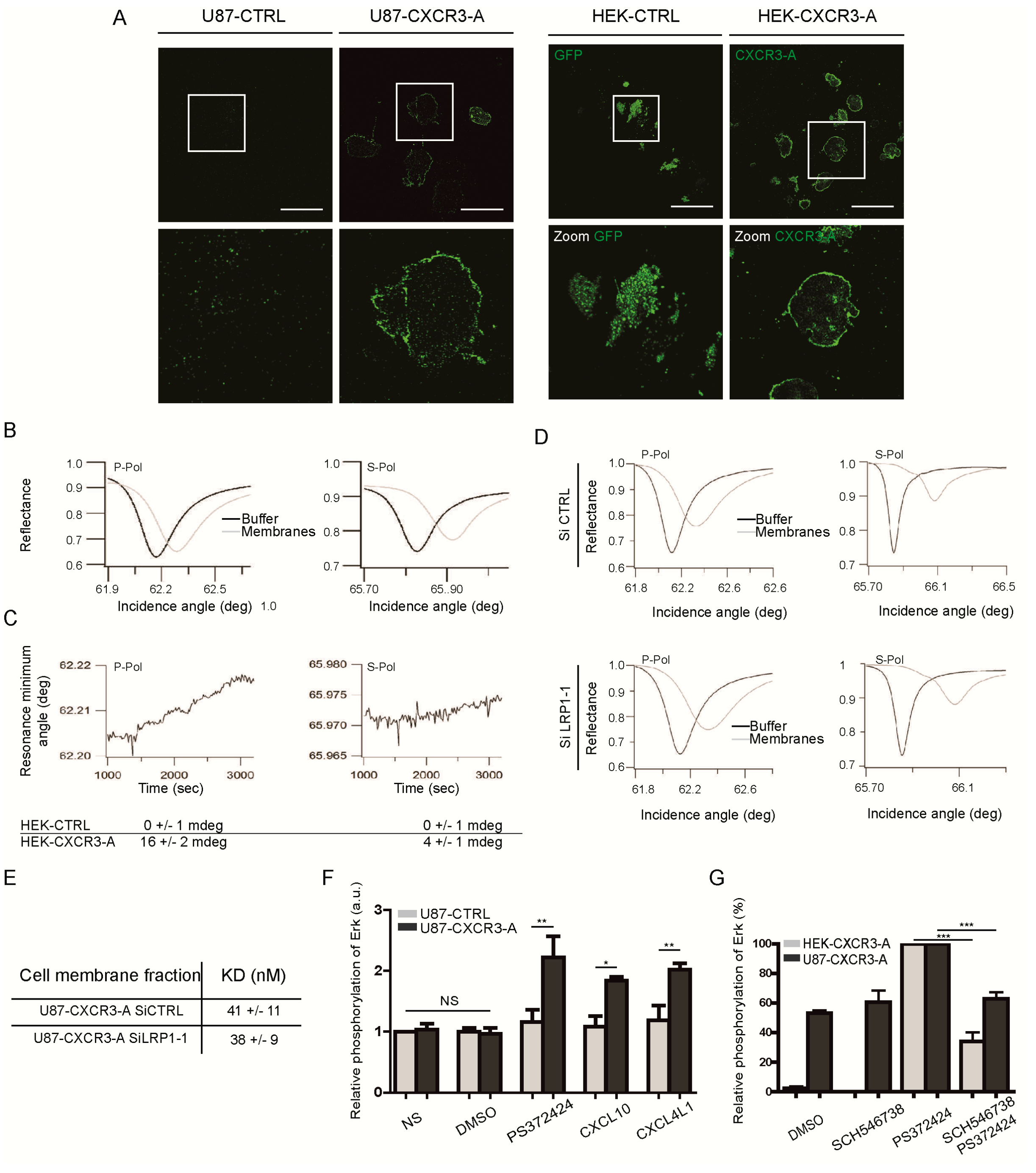

Supplementary Fig. 3: Conformational, dynamic and activation properties of CXCR3-A

(A) Caption on the PWR prism of U87-CRTL/U87-CXCR3-A and HEK-CTRL/HEK-CXCR3-A cell fragments. Sections were observed at 630× magnification under a confocal laser-scanning microscope (Nikon eclipse Ti) and images were acquired with the NIS Image browser software. Scale: 5µm. (B) PWR spectra for the PWR sensor before and after immobilization of the cell membrane fragments obtained with p- (left panel) and s- (right panel) polarized light. (C) Changes in the minimum resonance position following incremental addition of PS372424 to the cell fragments, as a function of time obtained with p- (left panel) or s-(right panel) polarized light. (D) Spectral shifts observed after deposition of U87-CXCR3-A cell fragments pre-treated with SiRNA CTRL (upper panel) and SiRNA LRP1 (lower panel) on the PWR sensor before and after immobilization of the cell membrane fragments. (E) KD values for PS372424 interaction with CXCR3-A in U87-CXCR3-A treated with siRNA CTRL or siRNA LRP1. (F) Relative pERK quantification in U87-CXCR3-A cells stimulated with CXCR3

agonists (100 ng/ml: PS372424, CXCL10 or CXCL4L1) (western blot in figure 2G). Values normalized to those obtained for U87-CTRL in non-stimulated condition. NS: non-stimulated condition. a.u.: arbitrary unit. *** P<0.001, ** P<0.01, * P<0.05. (G) Relative pERK quantification in U87-CXCR3-A and HEK-CXCR3-A cells pretreated or not with the antagonist SCH546738 (2.2nM) and stimulated with PS372424 (western blot in figure 2H). Values normalized to those obtained for DMSO condition. a.u.: arbitrary unit. *** P<0.001. The experiments have been repeated 3 times with identical results. Multiple comparisons were performed with one-way analysis of variance, followed by Bonferroni post hoc tests.

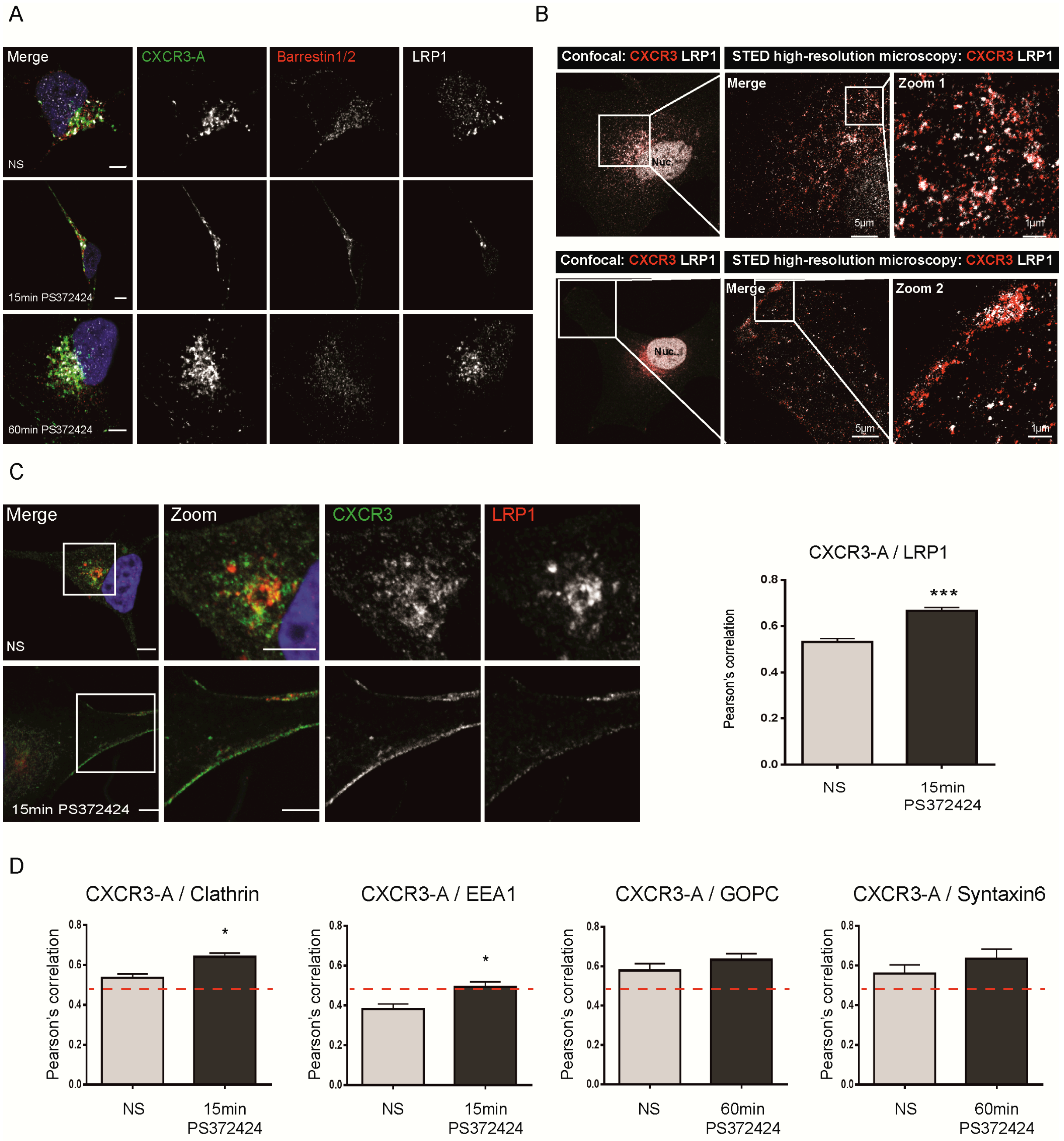

Supplementary Fig.4: CXCR3-A internalization and trafficking and CXCR7 expression

(A) Membrane or perinuclear localization of CXCR3 (λ=488nm) and colocalization with β-arrestin1/2 (λ=547nm) were shown in non stimulated (NS) HEK-CXCR3-A cells and upon stimulation at 15 and 60 minutes. LRP1 immunostaining (λ=647nm). (B) STED high resolution miscoscopy with their correlated confocal images. (C) Colocalization of CXCR3 and LRP1 shown by confocal microscopy (CXCR3 λ=488nm, LRP1 λ=547nm) in U87-CXCR3-A cells. Scale: 5 µm, NS: non-stimulated condition, 15min agonist: stimulated condition. 630× magnification (Nikon eclipse Ti confocal laser-scanning microscope). Pearson's correlations were calculated using NIS-Element AR 64-Bit software. All the results from three independent experiments were combined to calculate mean and SEM, and values were normalized to those obtained for the control cells (n=30), *** P<0.001. (D) Internalization and recycling of CXCR3 in U87-CXCR3-A were studied by immunostaining using antibodies against clathrin, EEA1, GOPC or Syntaxin6. Colocalizations were performed using NIS-Element AR 64-Bit software to calculate the Pearson's correlation. The results from three independent experiments

were combined to calculate mean and SEM (n=30), * $P<0.05$. Pearson's analyses were performed directly on NIS-Element AR 64-Bit software. Statistical comparison between two groups was performed using the Mann-Withney test.

A

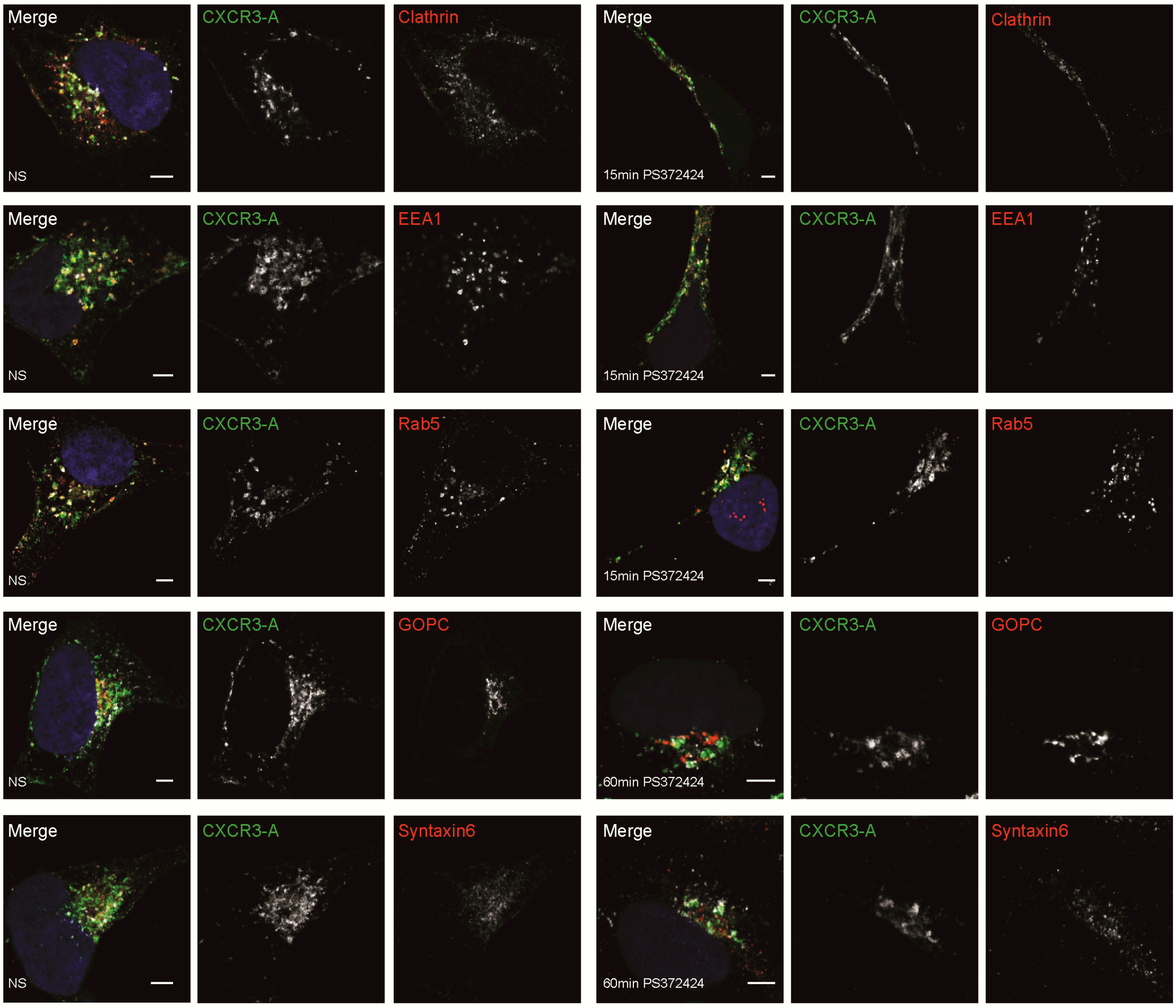

B

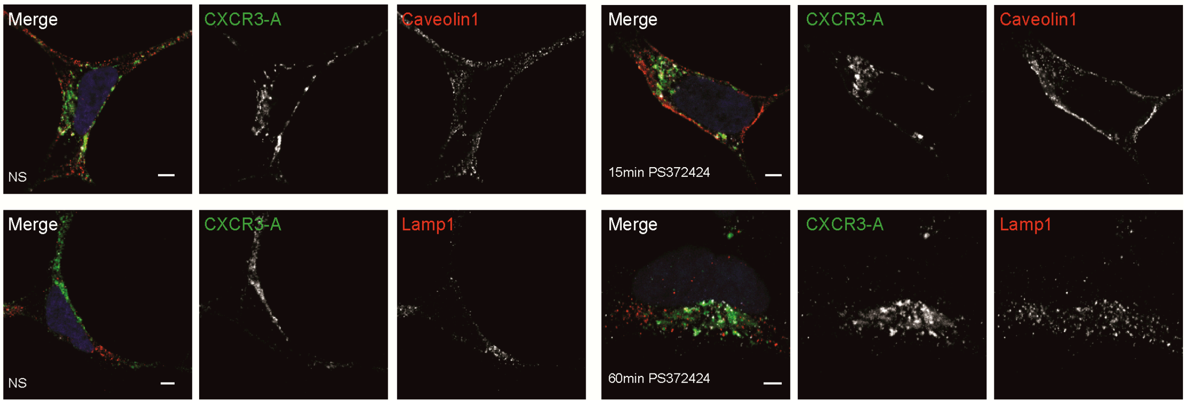

Supplementary Fig.5: CXCR3-A internalization and trafficking in HEK-CXCR3-A cells

(A) Membrane or perinuclear localization of CXCR3 (λ=488nm) and colocalization with LRP1 (λ=647nm) were shown in non-stimulated (NS) cells and upon stimulation with CXCR3 agonist at 15 and 60 minutes. Internalization and recycling of CXCR3 were studied by immunostaining using antibodies against clathrin, EEA1, Rab5, GOPC or Syntaxin6 (for each marker λ=547nm). Scale: 5µm. Sections were observed at 630× magnification under a confocal laser-scanning microscope (Nikon eclipse Ti) and images were acquired with the NIS Image browser software. (B) CXCR3-A

internalization into early endosome is caveolin-independent and is not degraded in lysosomes. No colocalization was seen between CXCR3-A and caveolin1 in non-stimulated or at 15min of stimulation in HEK-CXCR3-A cells. No colocalization was seen between CXCR3-A and Lamp-1 in non-stimulated or at 60 min of stimulation in HEK-CXCR3-A cells. Sections were observed at 630× magnification under a confocal laser-scanning microscope (Nikon eclipse Ti). Scale: 5 μm. The experiments have been repeated 3 times with identical results.

A

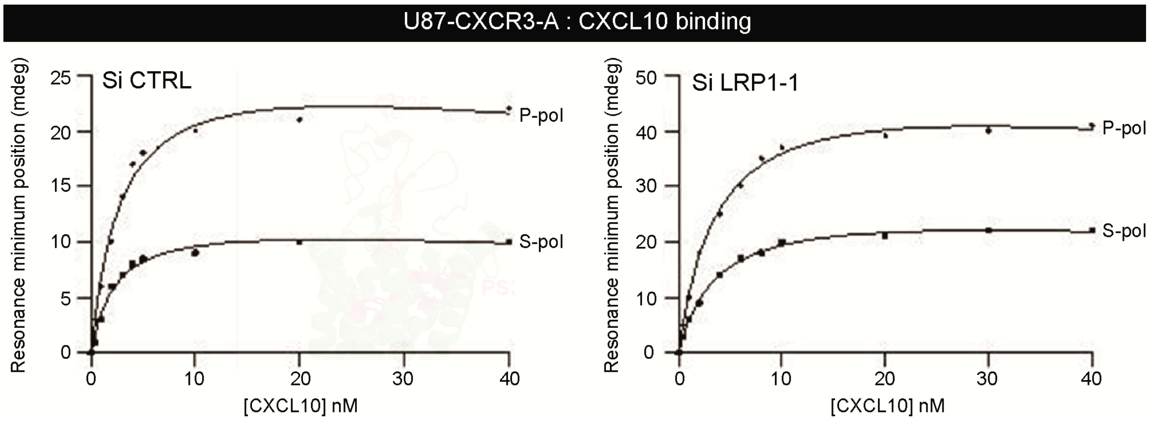

Cell membrane fraction	PWR spectral shifts (mdeg)		KD (nM)
	P-pol	S-pol	
U87-CTRL Si CTRL	0.5 +/- 1	1 +/- 1	No binding
U87-CTRL Si LRP1-1	1.5 +/- 1	0.5 +/- 0.5	No binding
U87-CXCR3-A Si CTRL	25 +/- 4	9 +/- 3	2.9 +/- 0.8
U87-CXCR3-A Si LRP1-1	40 +/- 7	25 +/- 5	3.2 +/- 0.7

B

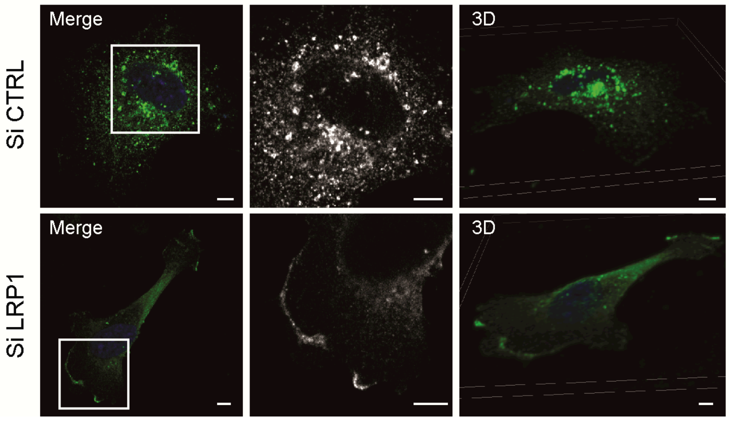

Supplementary Fig.6: LRP1 downregulation regulates CXCL10 binding and CXCR3 internalization
(A) Binding of CXCL10 to U87-CXCR3-A cells – effect of siRNA LRP1 treatment. Resonance shifts observed and KD values: PWR spectral changes induced by CXCL10 binding to the CXCR3-A and dissociation constants. U87 cell membrane fragments expressing the CXCR3-A were treated with siRNA CTRL and siRNA LRP1. (B) 3D representation of CXCR3-A distribution along the membrane or perinuclear with siRNA CTRL or siRNA LRP1. CXCR3 immunostaining in U87-CXCR3-A cells treated with Si CTRL or 3 different siRNA against human LRP1. Scale: 5 µm. The experiments have been repeated 3 times with identical results.

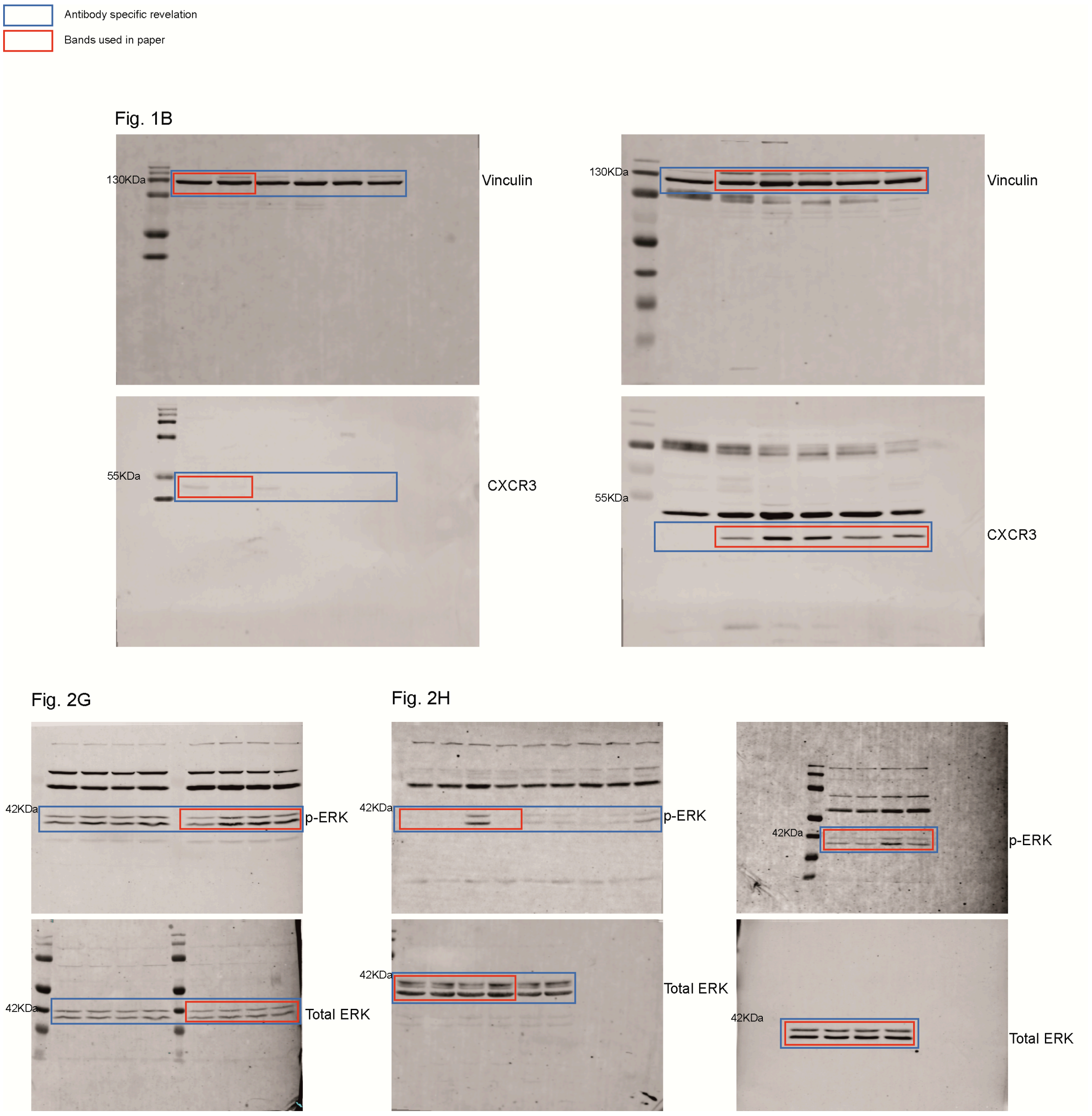

Supplementary Fig.7: Uncropped immunoblot

Antibody specific revelation

Bands used in paper

Fig. 3B

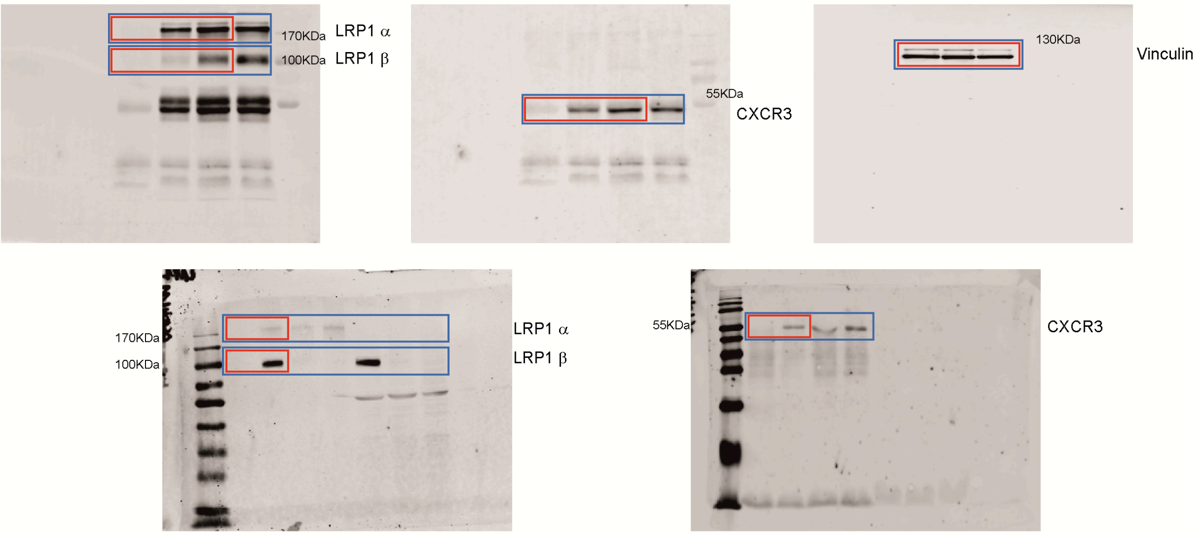

Fig. 3C

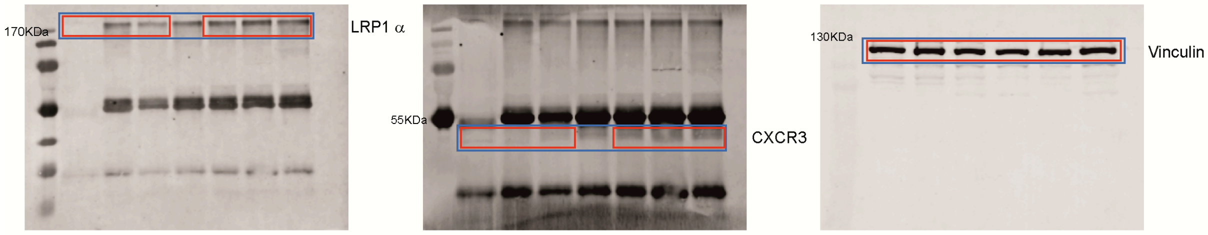

Fig. 3D

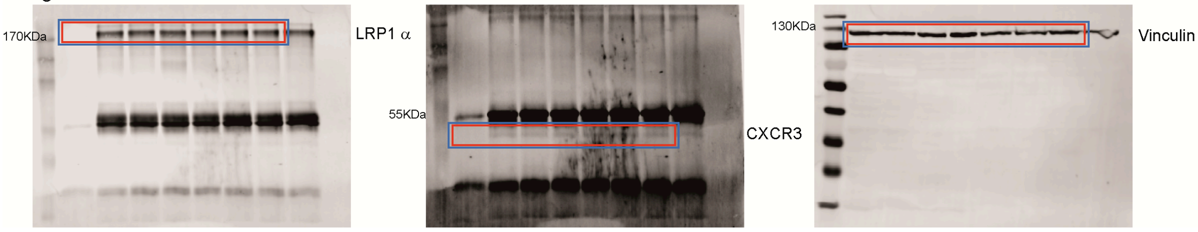

Supplementary Fig.8: Uncropped immunoblot

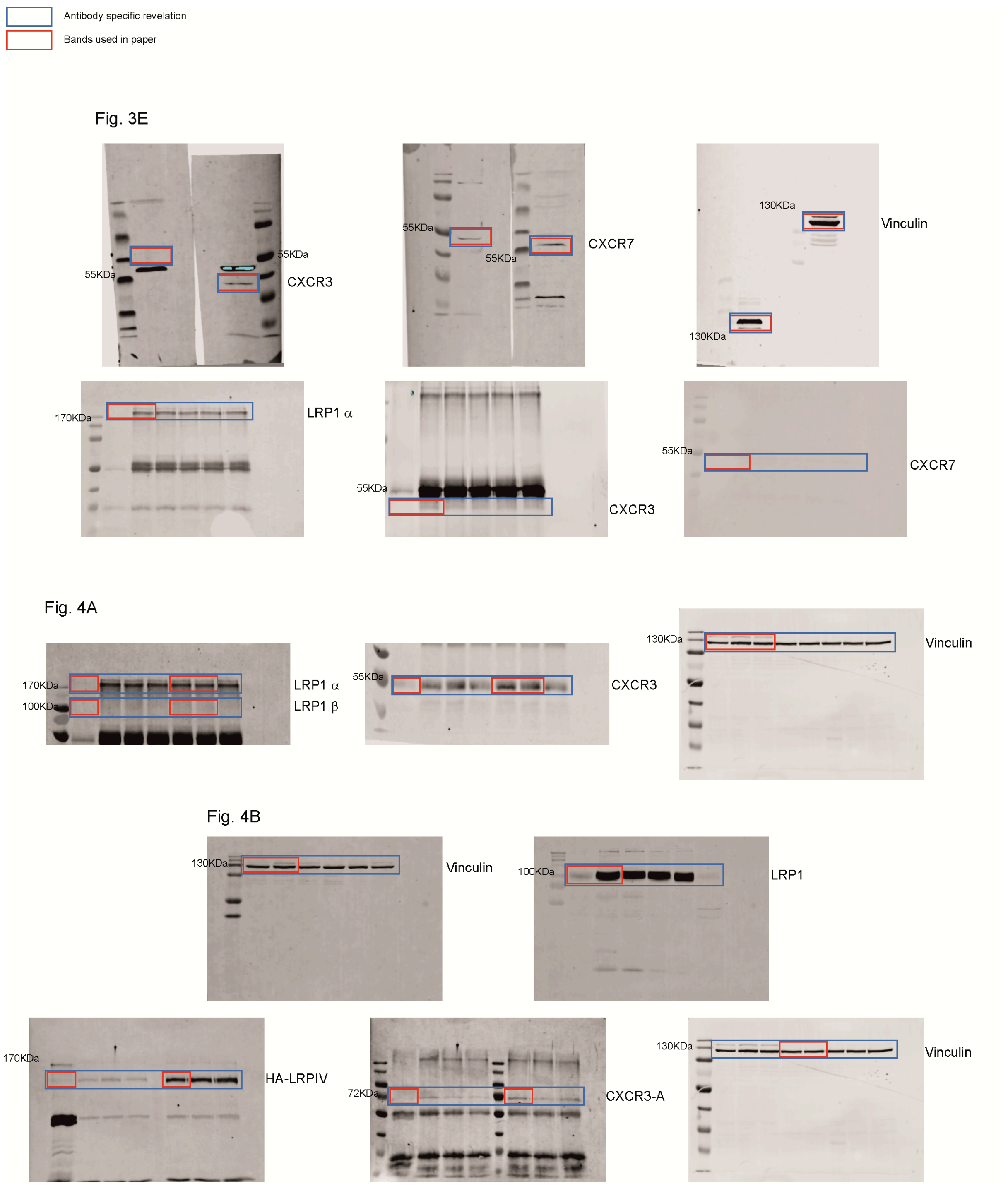

Supplementary Fig.9: Uncropped immunoblot

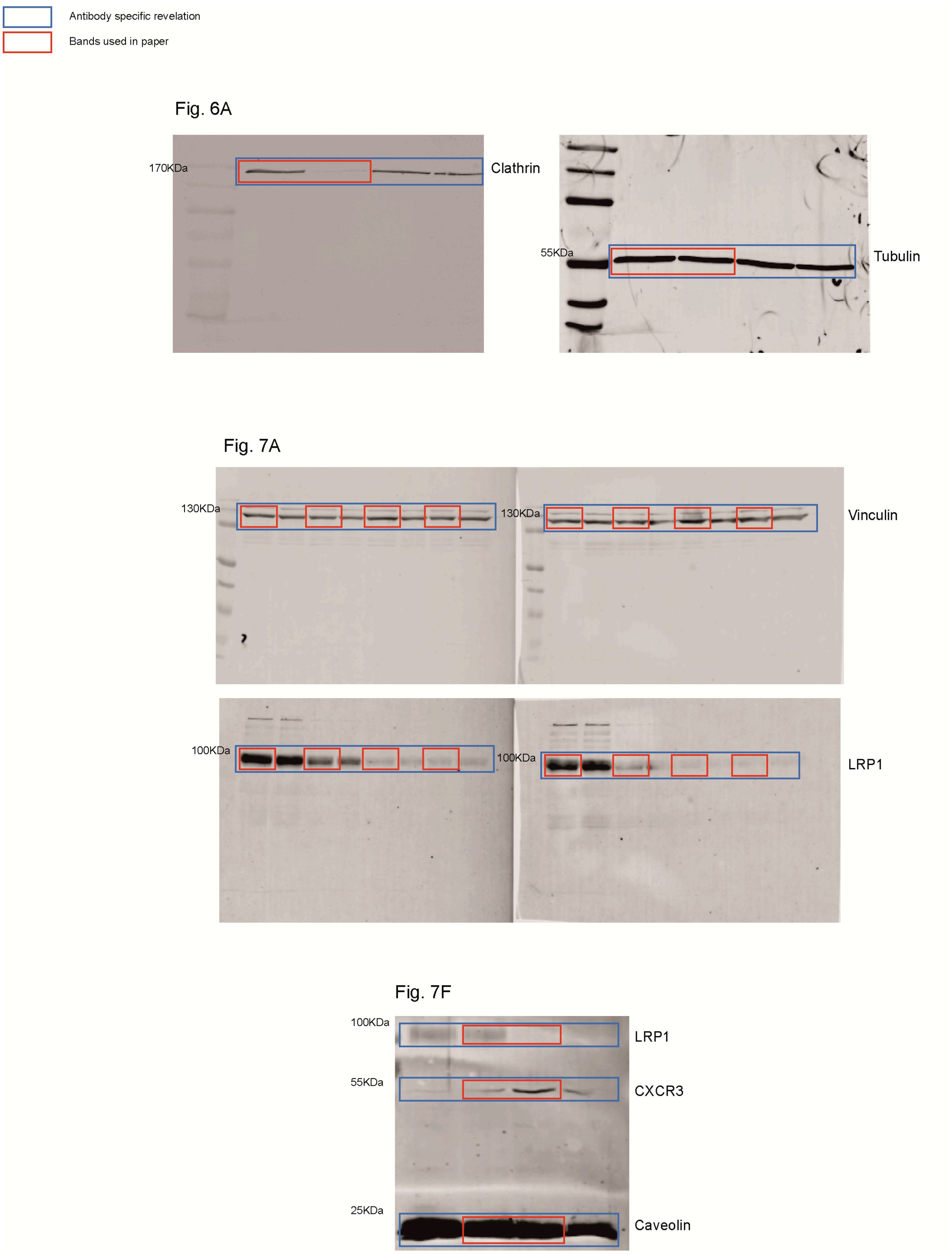

Supplementary Fig.10: Uncropped immunoblot

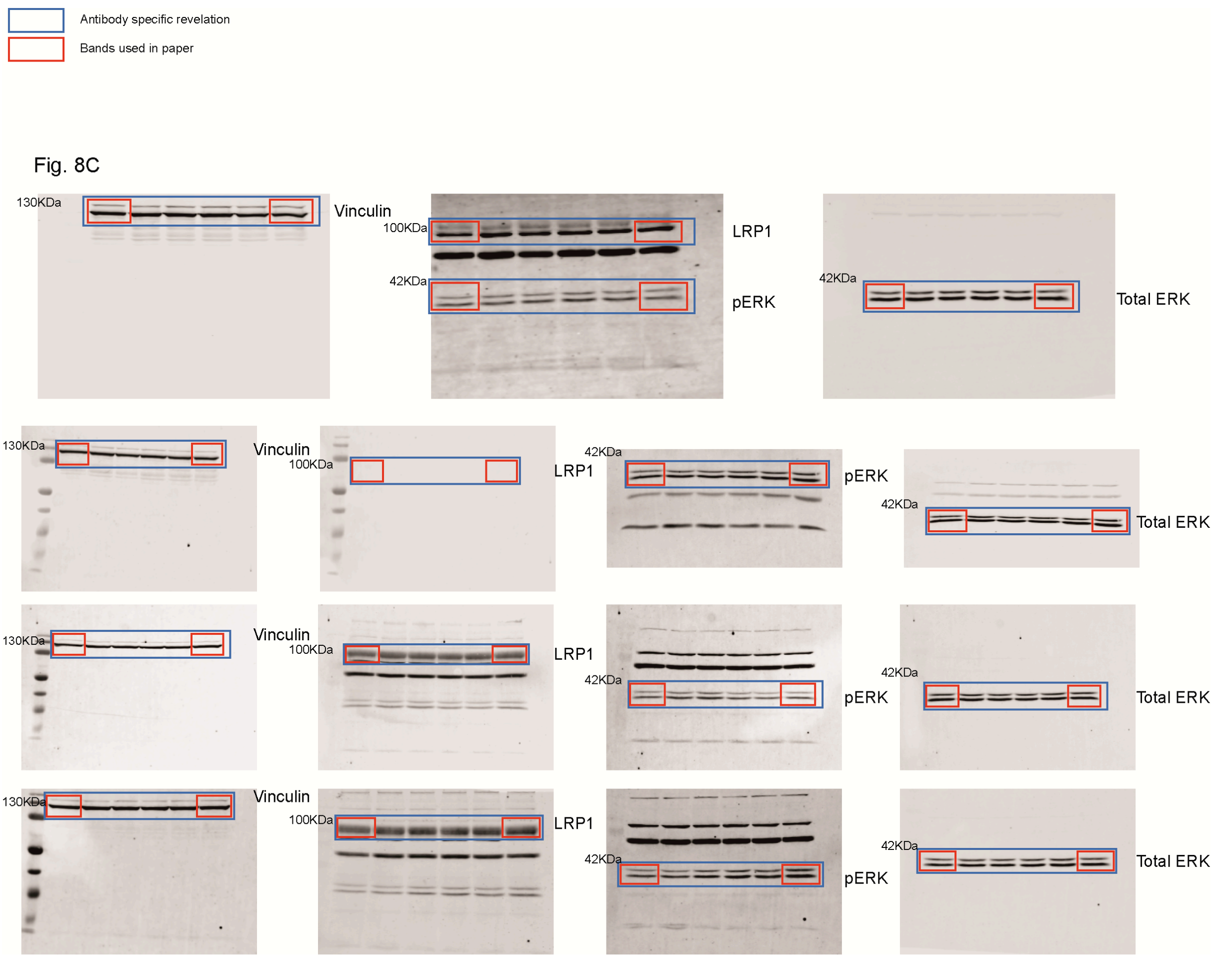

Supplementary Fig.11: Uncropped immunoblot

SUPPLEMENTARY REFERENCES

1 Allen, M., Bjerke, M., Edlund, H., Nelander, S. & Westermark, B. Origin of the U87MG glioma cell line: Good news and bad news. *Science translational medicine* **8**, 354re353, doi:10.1126/scitranslmed.aaf6853 (2016).

2 Ponten, J. & Macintyre, E. H. Long term culture of normal and neoplastic human glia. *Acta pathologica et microbiologica Scandinavica* **74**, 465-486 (1968).

3 Sasai, K., Akagi, T., Aoyanagi, E., Tabu, K., Kaneko, S. and Tanaka, S. O6-methylguanine-DNA methyltransferase is downregulated in transformed astrocyte cells: implications for anti glioma therapies. Mol Cancer. 6, 36 (2007)

4 Stein GH. T98G: an anchorage-independent human tumor cell line that exhibits stationary phase G1 arrest in vitro. J Cell Physiol. 99:43-54 (1979).
